# Community-Based Pilot Study of *Chlamydia trachomatis* and *Neisseria gonorrhoeae* Urogenital Infections Among Young Adults in the North and the Central Coast of Portugal

**DOI:** 10.3390/medicina61101749

**Published:** 2025-09-25

**Authors:** Rafaela Rodrigues, Sónia Loureiro, Inês João, Inês Jordão, Maria José Borrego, Carlos Catalão, Ana Rita Silva, Carlos Sousa, Nuno Vale

**Affiliations:** 1 PerMed Research Group, Center for Health Technology and Services Research (CINTESIS), Rua Doutor Plácido da Costa, 4200-450 Porto, Portugal; rafaela24sofia@hotmail.com; 2 CINTESIS@RISE, Faculty of Medicine, University of Porto, Alameda Professor Hernâni Monteiro, 4200-319 Porto, Portugal; 3 Molecular Diagnostics Laboratory, Unilabs Portugal, Centro Empresarial Lionesa Porto, Rua Lionesa, 4465-671 Leça do Balio, Portugal; sonia.santos.loureiro@unilabs.com (S.L.); ana.rita.silva@unilabs.com (A.R.S.); carlos.sousa@unilabs.com (C.S.); 4 National Reference Laboratory (NRL) for Sexually Transmitted Infections (STI), Department of Infectious Diseases, National Institute of Health Doutor Ricardo Jorge, 1649-016 Lisboa, Portugal; ines.joao@insa.min-saude.pt (I.J.); m.jose.borrego@insa.min-saude.pt (M.J.B.); 5 Department of Medical Sciences, University of Aveiro, 3810-193 Aveiro, Portugal; inesvjordao@ua.pt; 6 Roche Sistemas de Diagnósticos, Estrada Nacional 249-1, 2720-413 Amadora, Portugal; carlos.catalao@roche.com; 7 Department of Community Medicine, Health Information and Decision (MEDCIDS), Faculty of Medicine, University of Porto, Rua Doutor Plácido da Costa, 4200-450 Porto, Portugal

**Keywords:** sexually transmitted infections, chlamydial infections, gonococcal infections, dual-screening, *ompA*-genotyping

## Abstract

*Background and Objectives*: *Chlamydia trachomatis* (CT) and *Neisseria gonorrhoeae* (NG) are among the most common sexually transmitted infections (STIs), with the highest incidence in individuals aged up to 25 years. However, data from Portugal remain scarce. This cross-sectional pilot study aimed to assess the prevalence of urogenital CT and NG infections in young adults in Portugal and to identify the major CT genotypes circulating in this population. *Materials and Methods*: A total of 152 young adults aged 18–25 years were recruited over a six-month period from universities, a sports club, and a coworking space. Urine samples were tested using the Cobas 4800 CT/NG assay (Roche, Rotkreuz, Switzerland). CT-positive samples were further genotyped based on ompA gene diversity to identify major urogenital genotypes (D to K). All participants provided informed consent and completed a questionnaire on demographic characteristics and risk behaviors. *Results*: Of the 152 urine samples analyzed, five tested positive for CT, one for NG, and one sample showed co-infection with both pathogens. None of the participants with positive results reported symptoms. Genotyping of CT-positive samples identified ompA genotype E in three cases. *Conclusions*: Despite limitations such as small sample size and convenience sampling, this pilot study offers preliminary insights into the prevalence and genetic diversity of CT and NG infections among young adults in Portugal. The findings highlight the need for expanded screening programs, site-specific sample collection, and culture-based diagnostics to support evidence-based public health strategies targeting these infections.

## 1. Introduction

*Chlamydia trachomatis* (CT) is an obligate intracellular pathogen, depending on the host cells for its replication, which is responsible for causing chlamydial infections [1]. On its cell cycle of infection, this pathogen alternates between two distinct stages: the elementary body (EB), which is an infectious form, and the metabolically active and non-infectious form, the reticulate body (RB) [2,3]. In contrast, *Neisseria gonorrhoea* (NG), which is the causative agent of gonococcal infections, initiates infection with an interaction with the host cell, facilitated by the recognition of specific host cell receptors, such as CD46 and CR3, and communication via type IV pili. Although a primary immune action succeeds in eliminating the pathogen, predominantly through neutrophil-mediated phagocytosis, NG often survives, leading to the persistence of the infection [4]. Both of these pathogens are causative of the most common bacterial sexually transmitted infections (STIs), namely in Europe, and they are of particular interest due to their similar manifestations and outcomes [5,6,7].

**Figure 1:**
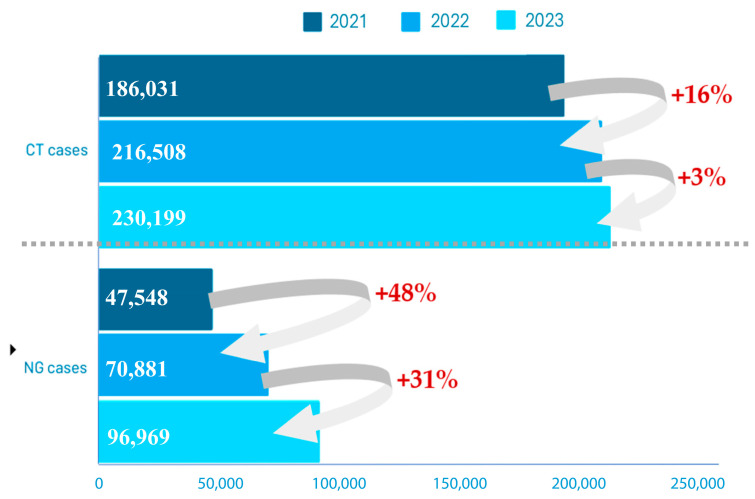
Reported cases of *Chlamydia trachomatis* (CT) and *Neisseria gonorrhoeae* (NG) infections in EU/EEA countries from 2021 to 2023. Numbers represent total reported cases per year, and arrows indicate the relative increase compared to the previous year. Data adapted from ECDC [6,7,11,12,13].

STIs pose a significant challenge in the field of public health and represent one of the foremost contemporary issues worldwide, even though the majority of them are curable or can be controlled with adequate treatment [8]. According to the European Centre for Disease Prevention and Control (ECDC), recent data show evidence regarding increased numbers of STIs, especially gonorrhea and chlamydia, in the European Union/European Economic Area (Figure 1) [5]. STIs significantly impact morbidity, affecting individuals’ quality of life, particularly in terms of sexual, psychological, and reproductive health [4,9,10].

Women are considered to be more affected by this issue than men, primarily due to their anatomy. The female reproductive system is more vulnerable because it is more exposed, making it easier for pathogens to invade. In fact, the cervix is the most affected anatomical region by CT and NG [14]. However, these bacteria can also infect the anorectal and oropharyngeal tracts, according to sexual intercourse habits [4]. Although the primary mode of transmission is sexual contact, vertical transmission is also possible [15]. Perinatal transmission of CT and NG infections can lead to neonatal complications such as conjunctivitis, nasopharyngeal infections, ocular discharge, swollen eyelids, and, in severe cases, sepsis [16].

CT and NG co-infection, or co-infection with other pathogens, namely human papillomavirus and *Mycoplasma genitalium*, is considered common [17,18,19]. Contrary to NG, which, in men, typically presents as urethritis, genital CT infection is mostly asymptomatic (in approximately 70–95% of cases in women and over 50% in men), which may contribute to the development of clinical complications that are not detected in time, as well as to uninterrupted transmission [20].

The worst sequelae of CT and NG infections are pelvic inflammatory disease, chronic pain, acute epididymitis, salpingitis, ectopic pregnancy, and infertility, which are also associated with higher medical treatment costs [21,22]. Importantly, the literature also suggests a possible association between persistent CT infection and neoplasia development [23,24,25].

This evidence highlights the need for the development of strategies to combat this preventable public health problem. Indeed, preventing CT and NG infections should be the primary goal, through primary prevention measures, as both infections are preventable and cost-effective to address. Specifically, governmental and health entities should invest in initiatives such as educational awareness campaigns in schools and within the community. Beyond these primary prevention strategies, secondary prevention is also crucial, encompassing early detection and prompt treatment through screening programs. Accurate screening of asymptomatic individuals is one of the most effective methods to fight against CT and NG infections, as it enables timely treatment, helps prevent antibiotic resistance, and breaks the chain of transmission [26]. When detected early, these bacterial infections can be easily treated with antibiotics [4,26]. Of note, although screening of asymptomatic individuals is considered a cornerstone of public health strategies against these infections, in clinical and research settings, both asymptomatic and symptomatic individuals are relevant and should both be included.

The molecular characterization of CT can provide valuable insights on the genetic characteristics of circulating strains, which would provide better understanding of their distribution, spread, pathogenicity, and potential differences in epidemiology [27]. In detail, CT *ompA* genotyping is based on the variability in the *ompA* gene encoding for the major outer membrane protein (MOMP), and the following 15 major *ompA* genotypes have been defined: A, B/Ba, C (the causative agent of trachoma), D/Da, E, F, G/Ga, H, I/Ia, J/Ja, K (associated with sexually transmitted infections and vertical transmission (mother to child)), and L1, L2, L2b, and L3 (responsible for lymphogranuloma venereum (LGV)) [27,28,29,30,31,32,33,34,35,36].

Previous studies in this field conducted in Portugal are reports focused on specific risk groups, potentially leading to overestimated prevalence rates [35]. Moreover, given increasing migratory flow, with immigrants constituting approximately 10% of the population, it will become imperative to recognize that existing data may be outdated [36].

Therefore, the present pilot study focuses on CT and NG sexually transmitted urogenital infections (serovars D–K) in Portugal. Using a non-invasive urine-based screening method, this study aims to explore the feasibility of estimating the prevalence of CT and NG infections among young adults, aged 18–25 years, who had already initiated sexual activity, regardless of symptom presentation. In addition, ompA genotyping of CT-positive samples is performed to provide preliminary insights into genetic diversity and potential geographic distribution. Given the limited sample size, this pilot study is not intended to provide definitive prevalence estimates or genotype distribution across regions, but rather to demonstrate the feasibility and need for such screening, providing preliminary data to support the expansion of future studies across Portugal.

## 2. Materials and Methods

### 2.1. Study Design

Cross-sectional pilot study developed during the first six months of 2024. The institutions and companies involved in this study were selected by convenience sampling and invited to participate. Upon study approval, information about the screening was disseminated internally through posters, flyers, and direct communication within each setting. No incentives were provided to participants. Young adults aged 18–25 years, who had already initiated their sexual activity, were eligible to participate, regardless of whether or not they presented symptoms of these STIs. Participation rates were not formally recorded, as this was an exploratory pilot study.

### 2.2. Clinical Specimens and Sample Size

This study included a total of 152 specimens for CT and NG testing, collected at distinct points of the north central coast of Portugal, from participants who had initiated their sexual life and were aged 18–25 years old, with or without infection symptoms).

Herein we have used self-collected urine specimens, a type of sample which potentially increases the success of adhesion. In fact, we opted to collect non-invasive urine samples due to the easier recruitment of participants, particularly for a population in which invasive methods such as urethral scraping or prostate massage could result in even lower participation rates. While we acknowledge that these invasive methods may increase the rate of CT positivity, mainly in women, our decision was based on balancing test efficacy with the feasibility of participant recruitment.

**Figure 2:**
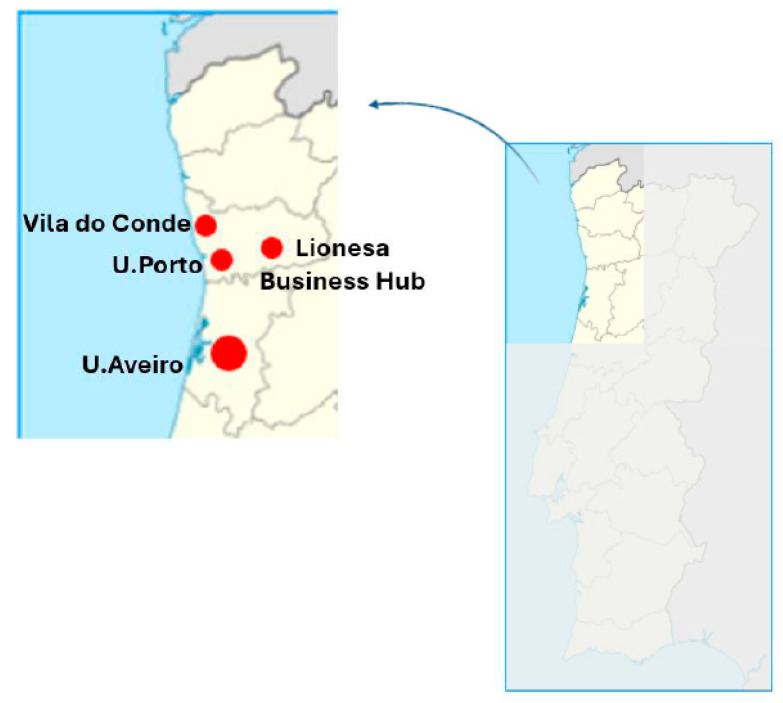
Recruitment sites along the north central coast of Portugal. Participants were recruited from Vila do Conde (Rio Ave F.C.), U. Porto, U. Aveiro, and Lionesa Business Hub. On the right, the continental territory of Portugal is shown, with the north and central coast highlighted. The left panel provides a zoomed-in view of this region, indicating all recruitment sites. Map showing recruitment zones is a figure created by the authors using BioRender (available from: https://www.biorender.com/).

The participants were enrolled from the following institutions: Rio Ave F.C.; University of Porto; Lionesa Business Hub of Porto; and University of Aveiro (Figure 2). Importantly, participants could enroll in our study regardless of whether they were asymptomatic or symptomatic.

This study was approved by the ethics committee of the Faculty of Medicine of the University of Porto (69/CEFMUP/2022); therefore, the method chosen to safeguard the identity and privacy of participants was the random assignment of unique anonymity codes attributed on the date of collection, which allowed the participant themself to identify their respective test results. Concomitantly, the participants had to sign an informed consent form and fill out a Q&A (choosing a Portuguese or English version) focusing on baseline characteristics, risk factors, history of infections, and genital symptoms (Table 1). In detail, the questions included the following: age, age at first sexual intercourse, sex, sexual orientation, nationality, if the participant was sexually active, number of sex partners at present and during life, condom use, STIs at present or in the past and which one(s), and symptoms presented. From each participant, a questionnaire and a urine sample were identified with the same anonymous code. Depending on the place of a specimen’s collection, urine samples were proceeded according to the manufacturer’s instructions, being placed immediately, or 15 h later (stored in ice in the meantime), in the laboratory, into Cobas PCR media, contained in the urine sample kit (Roche Molecular Systems). All samples were then submitted to the Molecular Biology Laboratory at Unilabs, located in Leça do Balio, Porto, Portugal, to be stored at 4 °C for up to three months before being tested. The specimens were collected over a 6-month period, from January 2024 to June 2024.

Of note, to guarantee anonymity, all participants could contact via email, provided in the informed consent document, one to three months after the collection date, to request the clinical report of their results.

### 2.3. Cobas 4800 Workflow

Currently, nucleic acid amplification tests (NAATs) are the gold standard for screening and diagnostics, due to their high sensitivity and specificity, and there are several commercially available tests [1]. In this work, we used the Cobas^®^ 4800 CT/NG test, conducted at the Molecular Biology Unilabs Laboratory, Porto, Portugal, according to the manufacturer’s instructions. This test has a high sensitivity for detecting CT and NG cases using urine specimens, around 96–99% for both infections; therefore, it ensures that most infected individuals are correctly identified by the test. The test specificity is also high for CT and NG detection, 98–100% and 99–100%, respectively, meaning that is effective at correctly identifying individuals who are not infected, minimizing the number of false-positive results [37,38].

Briefly, when using Cobas^®^ 4800 for the CT/NG testing, the run comprised 22 samples and could be performed in approximately 2.75 h, including the maintenance step. Of note, to guarantee the quality control of the process, one set of Cobas^®^ CT/NG Test Positive and Negative Controls were included in each run. This equipment allows for the detection of both bacteria using a dual-target approach [39]. In detail, to detect CT, c4800 uses primers, CTMP101 and CTMP102, that define a sequence of 182 nucleotides in the chromosomal DNA, specifically in *ompA*, and CP102 and CP103, which define a sequence of 206 nucleotides within the cryptic plasmid. Similarly, to detect NG, the primers used by c4800 are NG514 and NG519, which define a sequence of 190 nucleotides from the region DR-9a, a highly conserved direct-repeated region of the bacterium, and NG552 and NG579, to define a second region identified as a conserved sequence variant from this same region [40,41].

### 2.4. ompA Genotyping

This particular step of our project was carried out at the Portuguese National Reference Laboratory for Sexually Transmitted Infections, NRL, hosted at the National Institute of Health (NIH) Doutor Ricardo Jorge (Lisbon, Portugal). The DNA samples from all CT-positive specimens identified in our study were transported and subjected to *ompA* genotyping, as described elsewhere [28,42]. Briefly, PCR and nested PCR were performed using primers NLO/NRO and PCTM3/SERO2A, correspondingly, as described by Isaksson and colleagues [40,41]. Then, we performed an automated capillary electrophoresis on the QIAxcel Advanced system (Qiagen, Hilden, Germany) to evaluate the presence of the amplicons (~1014 bp). Samples with detectable PCR products were subsequently sequenced partially using primer ompA-1 (5′-TTA TGA TCG ACG GAA TTC T-3′), BigDye terminator V.1.1, and capillary sequencing (3130XL Genetic Analyzer; Applied Biosystems, Waltham, MA, USA) [42]. ompA genotypes were determined by BLASTn-based comparison using the ABRIcate tool against a custom database of reference and variant ompA sequences, following the method described by Lodhia and colleagues [28].

### 2.5. Data Analysis

Data from the questionnaires were transcribed to SPSS^®^ Statistics (version 26.0; SPSS Inc., Chicago, IL, USA) in order to create the database of this pilot study. Categorical variables were described using absolute and relative frequencies, *n* (%), while quantitative variables with normal distribution were described using the mean, standard deviation (SD), minimum (min), and maximum (max) values. Normal distribution was assessed through visual inspection of the histograms. All statistical analyses were performed using SPSS^®^ Statistics (version 26.0; SPSS Inc., Chicago, IL, USA). A significance level of α = 0.05 was used to determine statistical significance.

In order to understand the outcome (categorical variable) association with all the possible factors, different statistical analyses were conducted. For the factors that are continuous variables, a t-test was used; for the categorical variables, the chi-square test was used. Importantly, continuous variables were categorized in order to facilitate further analysis. In addition, a logistic regression analysis was applied to control for confounding factors and include multiple variables in a model, in order to gain insight into the factors that could explain variations in the outcomes (“having” or “not having” any of the infections under evaluation).

## 3. Results

### 3.1. Study Population

A total of 154 individuals participated in the screening test; notwithstanding, only 152 participants were eligible (two participants were excluded due to age). The study sample included 71 male and 80 female young adults, and one participant whose sex was not mentioned in the Q&A. Demographic and reproductive health characteristics are summarized in Table 1. The majority of participants were Portuguese (89.3%) and, among all participants, 84.6% stated that they were free of genitourinary symptoms at the time of screening.

Herein, 6 out of 152 individuals tested positive for CT infection. One of these also tested positive for NG, indicating a co-infection. Additionally, one participant tested positive for NG only. All positive individuals were aged 18–24 years and reported being asymptomatic at the time of screening. The co-infected individual was male, heterosexual, and reported multiple lifetime sexual partners, as well as a history of syphilis. The NG-only-positive case was female, bisexual, and of non-Portuguese nationality. Among the remaining five CT-positive participants, three were female (one heterosexual; two did not disclose sexual orientation) and two were male (one homosexual, one heterosexual).

To explore potential risk factors associated with both infections, we attempted exploratory univariable logistic regressions including demographic and behavioral variables. However, given the very low number of positive cases (*n* = 6 CT; *n* = 2 NG), the models produced unstable or non-estimable results (e.g., zero cells, inflated odds ratios, wide confidence intervals), and no statistically significant associations were identified. For transparency, the full exploratory regression outputs are provided in Appendix A, Table A1, but results are not suitable for inference.

### 3.2. CT Genotyping

**Figure 3:**
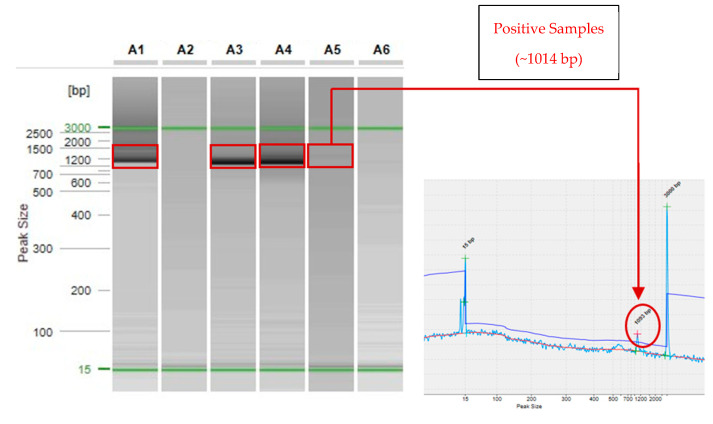
Analysis of PCR products from CT-positive samples (Lanes A1–A6) using automated capillary electrophoresis with QIAXCEL Advanced and ScreenGel Software (V2.1). OmpA amplicons (~1014 bp) are highlighted in red and are present in samples A1, A3, A4, and A5 (the latter showing a very faint band). On the right, the electropherogram of lane A5 is shown to confirm the presence of the ompA fragment. Green lines indicate the DNA alignment marker, containing fragments of 15 bp and 3000 bp. Samples A2 and A6 show no detectable amplicons.

From the six CT-positive samples, three were successfully ompA genotyped (50%) (Figure 3).

Two of the ompA-genotyped CT-positive samples were from men, whereas one was from a woman, all from individuals were aged 21–22 years. Table 2 includes detailed information per sequence, and nucleotide sequences are available at a public repository and GenBank, under the accession numbers PV106176, PV106177, and PV106178 [43].

Our preliminary results demonstrate that the only genotype detected was ompA genotype E (*n* = 3) (Table 2).

## 4. Discussion

Chlamydial and gonococcal infections are highly prevalent globally [44]. Therefore, it is important to establish screening to effectively control the dissemination of these infections.

In Portugal, there is no dedicated national screening program for CT and NG infections, and, consequently, no data on their prevalence. In fact, it is only since 2017 that the National System of Epidemiological Surveillance (SINAVE) has been implemented, which, although it does not permit the estimation of infection prevalence within the population, facilitates case reporting [45]. This system enables the transmission of data to the ECDC, where estimates for infection in Portugal are subsequently calculated [46]. Thus, to contribute to fulfilling this gap, we conducted an initial pilot study over a six-month period, with subsequent ongoing recruitment of participants to further increase the sample size, in different regions of Portugal. The study population included individuals from diverse national backgrounds, achieving approximately 10.5% immigrants (16 out of 152), closely reflecting the estimated 10% immigrant population in Portugal [36]. None of the immigrants tested positive for CT, while one tested positive for only NG.

This study’s limitations begin with the sampling strategy, which was constituted by a convenience sample involving nearby institutions (universities, a business hub, and a football club). This may not fully represent the broader young adult population in Portugal, introducing potential selection bias. While this community-based design was adequate for a pilot study, future research should include higher-risk populations and employ more diverse recruitment strategies. Additionally, we must acknowledge potential bias introduced by individuals who declined to participate, as these may include those who engage in risky behaviors and seek to benefit from free diagnostic services, as well as individuals who avoided participation due to fear of discovering an infection or confronting their sexual health status. Importantly, several participants chose not to answer all questions, which affected the statistical analysis of potential risk factors to an undetermined scale. Specifically, 1 participant refused to declare their sex at birth; 33 did not reveal their sexual orientation; 2 did not disclose their nationality; 3 did not mention if they had symptoms; and 1 did not indicate whether they had previously had an STI.

Despite the small sample size and all aforementioned limitations, which prevent accurate estimations of infection prevalence at the population level, 6 out of 152 specimens tested positive for CT, corresponding to a putative “prevalence” of 3.9% (95% CI: 1.5–8.3%), specific to this sample. Notably, one of these cases was also positive for NG. In total, two individuals tested positive for NG, corresponding to a putative “prevalence” of 1.3% (95% CI: 0.2–4.7%), again specific to this sample. Although no strong conclusions can be drawn and the findings cannot be generalized to estimate CT and NG prevalence at the population level, these sample-specific rates are in accordance with those reported in several European countries, following ECDC data [5,11,12,13].

Although only one case of CT and NG co-infection was detected, this finding highlights the importance of considering simultaneous screening for multiple STIs. Co-infections can complicate the clinical picture, as symptoms may overlap or be masked, potentially delaying diagnosis and treatment [47]. Furthermore, co-infected individuals may present a higher risk of onward transmission and may be more susceptible to long-term complications, such as pelvic inflammatory disease or infertility [48]. The identification of co-infection, even in a small sample, reinforces the need for integrated STI testing strategies, particularly in high-risk or underserved populations [49].

None of the participants who tested positive for CT or NG reported symptoms at the time of screening. This finding is consistent with the literature, as these infections are often asymptomatic. Furthermore, our statistical analysis did not identify any factor significantly associated with CT or NG positivity (Appendix A, Table A1). While this study lacked sufficient statistical power to detect such associations, this does not imply that the analyzed risk factors are unrelated to the infections; rather, the limited sample size prevented clarification of potential associations.

Methodologically, the use of urine samples to detect both bacteria has lower sensitivity compared with site-specific swabs, which may lead to underestimations of prevalence rates and impacts on epidemiological conclusions. While this method may not be ideal for clinical diagnosis, it was crucial for feasibility and participant compliance in this study. Urine-based NAATs offer good overall sensitivity and, due to their non-invasive nature, significantly improve acceptability and participation [37,38]. In this sense, although not the most sensitive method available, this approach was indispensable for generating preliminary epidemiological data in a population where such information is scarce.

In detail, the Cobas^®^ 4800 CT/NG test used in this study primarily detects urogenital chlamydial infections caused by serovars D–K, which are the most common and the focus of this research [38,39,41]. Urine samples were selected for their practicality and non-invasiveness, particularly suitable for young adults, a population that is very difficult to recruit. However, this approach does not detect infections at non-genital sites, such as anorectal or oropharyngeal regions, where CT and NG may reside asymptomatically [41]. In line with these limitations, future studies should consider collecting site-specific samples alongside urine specimens.

It is important to note that LGV serovars (L1–L3) mainly infect deeper genital and lymphatic tissues and are rarely present in urine, making them difficult to detect using this sample type [33,34]. Although ompA genotyping can differentiate LGV serovars, this was beyond the scope of our study, and prevalence estimates for LGV are therefore not provided [28].

NG detection was included due to overlapping clinical features, shared risk factors, and frequent co-existence with CT infections, taking advantage of the dual-target design of the assay used [4,38,39]. Indeed, a further limitation is that NG detection relied exclusively on molecular testing, without culture-based confirmation. While molecular assays offer high sensitivity and specificity and were appropriate for our primary aim of prevalence estimation, they do not allow for antimicrobial resistance monitoring. As NG culture remains crucial for resistance surveillance, it should be incorporated into future studies.

With regard to genotyping, only three of the six CT-positive samples were successfully genotyped, all identified as *ompA* genotype E. This limitation reflects the lower sensitivity of *ompA* genotyping compared to molecular detection assays such as the Cobas 4800 CT/NG, which employs a dual-target strategy (chromosomal and plasmid DNA) [40]. In contrast, genotyping relies on amplification of a single target and is more susceptible to failure in samples with low bacterial load, DNA degradation, or suboptimal specimen type (urine) [50]. Our genotyping failure rate (50%) was higher than previously reported, possibly due to transport conditions and the small number of positive cases [51,52]. The detection of genotype E aligns with previous data from Portugal and its predominance in most other countries [28,53].

In conclusion, despite the limitations, the chosen methodology was appropriate for the exploratory objectives of this project, providing preliminary insights into the prevalence of urogenital CT and NG infections in a community-based population. These findings highlight the need for broader screening strategies, site-specific sampling, and culture-based diagnostics in future research. Such steps will be essential to inform public health strategies and guide evidence-based prevention and control of CT and NG infections.

## 5. Conclusions

This pilot study has been instrumental in identifying key areas for further research and has underscored the urgent need to determine the prevalence of CT and NG infections in Portugal. The absence of a national screening program and the limited epidemiological data available highlight a critical gap in STI surveillance. By conducting this exploratory investigation, we have laid the groundwork for larger, more representative studies across different regions of the country, aiming to better understand the distribution of these infections within the Portuguese population.

The insights gained from this study, particularly the detection of asymptomatic infections and a case of co-infection, reinforce the importance of implementing broader and more inclusive screening strategies. These findings are aligned with the literature, supporting that CT and NG infections are circulating, silently, within the young adult population, with potential long-term health consequences if left undiagnosed and untreated. Therefore, the development of a national screening initiative, particularly targeting young and high-risk populations, is strongly recommended to enable early detection and timely intervention.

Moreover, the reluctance of some participants to disclose sensitive information or engage fully with the study further points to the necessity of public health campaigns that promote sexual health literacy, reduce stigma, and encourage active participation in STI screening.

In conclusion, these preliminary findings can inform policymakers and healthcare providers about the reality of these STIs in Portugal, supporting the development of evidence-based interventions and guiding the allocation of resources toward a more effective national response.

## Figures and Tables

**Table 1:** Baseline characteristics and demographics of the study population (*n* = 152).

Variable		Missing, *n* (%)
Sex, *n* (%)		1 (0.7)
Female	80 (53)	
Male	71 (47)	
Sexual Orientation, *n* (%)		33 (21.7)
Heterosexual	72 (60.5)	
Homosexual	19 (16)	
Bisexual	28 (23.5)	
Nationality, *n* (%)		2 (1.3)
Portuguese	134 (89.3)	
Other	16 (10.7)	
Age (years), x ± SD, min, max	21 ± 1.9, 18, 25	0 (0)
Age at first sexual intercourse (years), x ± SD, min, max	17.4 ± 2.3, 3, 23	1 (0.7)
Sexually active, *n* (%)		0 (0)
Yes	129 (84.9)	
No	23 (15.1)	
Number of sex partners currently, *n* (%)		11 (7.2)
0	22 (15.6)	
1	113 (80.1)	
>1	6 (4.3)	
Number of sex partners in life, *n* (%)		6 (3.9)
1	37 (25.3)	
>1	109 (74.7)	
Condom use, *n* (%)		0 (0)
Yes	62 (40.8)	
No	90 (59.2)	
STIs, *n* (%)		1 (0.7)
Yes	3 (2)	
No	148 (98)	
Symptoms, *n* (%)		3 (2)
Yes	23 (15.4)	
No	126 (84.6)	
Clusters, *n* (%)		0 (0)
U. Porto	31 (20.4)	
U. Aveiro	50 (32.9)	
Business Hub	25 (16.4)	
Rio Ave F.C.	46 (30.3)	

**Table 2:** Metadata of CT-positive samples through ompA genotyping method [43].

Sequencing ID/Sample ID	Type of Sample	Age	Sex	Sexual Orientation	Year Detected	A.C.E.	*ompA* Genotype
PV106178	Urine	21	F	NA	2024	+	E
204804129	Urine	20	M	Heterosexual	2024	−	−
PV106176	Urine	22	M	Heterosexual	2024	++	E
PV106177	Urine	21	M	Homosexual	2024	++	E
206219363	Urine	18	F	NA	2024	+F	−
215120440	Urine	24	F	Heterosexual	2024	−	−

A.C.E.: automated capillary electrophoresis qualitative evaluation; F: faint band visible in Figure 3 (Sample A5); +: Presence of PCR amplification products; -: PCR amplification products are not present; ++: Higher presence of PCR amplification products; NA: not answered.

## Data Availability

Not applicable.

## Appendix A

**Table A1:** Simple logistic regression analysis with CT and NG infections as dependent variables in the study population.

	CT			NG	
	OR [95% CI]	*p*-Value		OR [95% CI]	*p*-Value
Sex					
Female	Reference				
Male	1.13 [0.22; 5.79]	0.88		1.13 [0.07; 18.38]	0.93
Sexual Orientation					
Heterosexual	Reference				
Homosexual	1.28 [0.13; 13.03]	0.84		NE	1
Bisexual	NE	1		2.63 [0.16; 43.55]	0.50
Nationality					
Portugal	Reference				
Other	NE		1	0.11 [0.01; 1.89]	0.13
Age					
18–20	Reference				
21–24	1.72 [0.31; 9.72]	0.54		NE	1
25	NE	1		1	1
Age at first sexual intercourse					
<=14	Reference				
15–17	0.52 [0.05; 5.42]	0.58		NE	1
>=18	0.31 [0.03; 3.76]	0.36		1	1
Sexually active					
Yes	Reference				
No	NE	1		NE	1
Number of sex partners currently					
0	Reference				
1	NE	1		NE	1
>1	1	1		1	1
Number of sex partners in life					
1	Reference				
>1	1.73 [0.19; 15.31]	0.62		NE	1
Condom use					
Yes	Reference				
No	0.28 [0.03; 2.45]	0.25		NE	1
STIs					
Yes	Reference				
No	NE	1		NE	1
Symptoms					
Yes	Reference				
No	NE	1		NE	1
Clusters					
U. Porto	Reference				
U. Aveiro	0.61 [0.04; 10.16]	0.73		0.61 [0.04; 10.16]	0.73
Business Hub	2.61 [0.22; 30.57]	0.44		NE	1
Rio Ave F.C.	1.36 [0.12; 15.72]	0.80		NE	1

CT—*Chlamydia trachomatis*; NG—*Neisseria gonorrhoeae*; CI—confidence interval; OR—odds ratio; NE—non-estimable.
